# Atlas of telomeric repeat diversity in *Arabidopsis thaliana*

**DOI:** 10.1186/s13059-024-03388-3

**Published:** 2024-09-16

**Authors:** Yueqi Tao, Wenfei Xian, Zhigui Bao, Fernando A. Rabanal, Andrea Movilli, Christa Lanz, Gautam Shirsekar, Detlef Weigel

**Affiliations:** Department of Molecular Biology, Max Planck Institute for Biology Tübingen, Tübingen, 72076 Germany

**Keywords:** Telomere, Satellite repeats, Long reads, Arabidopsis

## Abstract

**Background:**

Telomeric repeat arrays at the ends of chromosomes are highly dynamic in composition, but their repetitive nature and technological limitations have made it difficult to assess their true variation in genome diversity surveys.

**Results:**

We have comprehensively characterized the sequence variation immediately adjacent to the canonical telomeric repeat arrays at the very ends of chromosomes in 74 genetically diverse *Arabidopsis thaliana* accessions. We first describe several types of distinct telomeric repeat units and then identify evolutionary processes such as local homogenization and higher-order repeat formation that shape diversity of chromosome ends. By comparing largely isogenic samples, we also determine repeat number variation of the degenerate and variant telomeric repeat array at both the germline and somatic levels. Finally, our analysis of haplotype structure uncovers chromosome end-specific patterns in the distribution of variant telomeric repeats, and their linkage to the more proximal non-coding region.

**Conclusions:**

Our findings illustrate the spectrum of telomeric repeat variation at multiple levels in *A. thaliana*—in germline and soma, across all chromosome ends, and across genetic groups—thereby expanding our knowledge of the evolution of chromosome ends.

**Supplementary Information:**

The online version contains supplementary material available at 10.1186/s13059-024-03388-3.

## Background

Telomeric repeat arrays are found at the termini of most eukaryotic chromosomes [1]. The very ends of the arrays, known as telomeres [2], commonly consist of canonical units with the formula (*T*)_*x*_(*A*)_*y*_(*G*)_*z*_ and act as functional caps that protect chromosome ends from degradation and fusion [3, 4]. These canonical repeats are being synthesized from an RNA template by telomerase, which ensures their sequence conservation [5]. In contrast to these highly conserved repeats, the immediately following sequences often include degenerate and variant telomeric repeats [6–9], which differ from the canonical unit in one or more base substitutions or small insertions and deletions (indels) [10]. The composition of the variant repeats displays remarkable heterogeneity within the same genetic group and among different chromosome ends [11–14], raising questions as to the evolutionary mechanisms that generate and maintain this diversity [15, 16]. This telomere-adjacent region serves as a transition zone between the telomere and the rest of the chromosome that contains genes and other genetic elements [1]. Specific types of variant telomeric repeats have been implicated in determining methylation state [17], protein binding [18], and formation of G-quadruplexes [19]. A comprehensive understanding of the evolutionary dynamics and functional significance of telomeres and telomere-adjacent regions must therefore begin with thorough knowledge of variation in the composition of telomeric repeats.

*Arabidopsis thaliana* has a seven-base-pair canonical unit TTTAGGG, which is the dominant telomeric unit in many other plant species as well [20, 21]. The presence of variant telomeric repeats in *A. thaliana* was first established with a yeast artificial chromosome strategy [8]. Subsequently, sequencing of PCR products revealed the heterogeneity of variant repeats from individual chromosome ends [22, 23]. Variant repeats have also been directly observed in unassembled sequencing reads [24], and they have been identified by partially assembling four chromosome ends in the Col-0 accession from Illumina short reads [17]. However, the highly repetitive nature of telomeric regions and the presence of identical sequences shared between repeat-adjacent regions, as well as large interstitial telomeric arrays in other parts of *A. thaliana* genomes, create ambiguity when mapping reads that are only hundreds base pairs long to specific positions of the genome [25–27]. As a result, variation in telomeric repeat content at *A. thaliana* chromosome ends remains largely uncharacterized and has been ignored in diversity studies.

New single-molecule sequencing methods, generating reads of more than 10 kilobases (kb) in length, which exceeds the size of full-length telomeric repeat tracts and extends into unique repeat-adjacent regions, can overcome the challenges of reconstructing full telomeric sequences [28]. However, although several *A. thaliana* genome assemblies have now been published [29–31], they have largely ignored the telomeric sequences apart from confirming that telomeres are structurally present at most chromosome ends. Pacific Biosciences High Fidelity (PacBio HiFi) sequencing is particularly well suited for reliable base calling in low-complexity telomeric repeats [32]. In addition, the circular sequencing mode of HiFi sequencing, wherein each DNA molecule is sequenced multiple times, allows us to confidently characterize somatic information such as repeat number variation in the telomeric regions, which is obscured in assemblies [33, 34].

In this study, we provide a high-resolution description of telomeric repeats for all ten chromosome ends in *A. thaliana*. We identify numerous types of variant telomeric repeats and previously undescribed sequence arrangement within the telomeric region, including higher-order repeats and inter-chromosomal similarity of non-telomeric fragments. We also investigate repeat number variation of non-canonical telomeric repeat arrays at both germline and somatic levels. We illustrate chromosome end-specific and genetic group-specific patterns of repeat haplotypes along with linkage disequilibrium between telomeric repeat arrays and their adjacent non-coding regions. Our findings significantly expand the collection of repeats derived from canonical telomeric repeats and telomeric sequence features in *A. thaliana*, setting the stage for a deeper understanding of the evolutionary mechanisms acting on them.

## Results

### Profiling telomeric regions in *A. thaliana*

To investigate the sequence content of telomeric regions, defined here as canonical telomeric repeats, adjacent variant and degenerate telomeric repeats as well as any unique sequences interspersed in these repeats, HiFi reads from 74 *A. thaliana* accessions of diverse geographic origins were used (Additional file 2: Table S1). Among them, 66 accessions were grouped into four main genetic clusters (Additional file 1: Fig. S1), with 43 non-relict accessions from Europe, 11 from Asia, 9 from Iberian relicts, and three from North America. Eight further accessions were from various relict groups [35–37].

For each accession, HiFi reads were unambiguously extracted for the eight non-ribosomal DNA (rDNA)-binding chromosome ends (Additional file 1: Fig. S2; Additional file 1: Fig. S3; Additional file 1: Fig. S4; Additional file 1: Fig. S5; Additional file 1: Fig. S6a). For the ends of the p-arms of chromosome 2 and 4 (hereafter, chr2p and chr4p), which remain incompletely assembled due to large 45S rDNA tandem arrays that are immediately adjacent to the telomeres [38], reads could be assigned to two groups but could not be precisely assigned to chr2p or chr4p (Additional file 1: Fig. S6b).

**Fig. 1:**
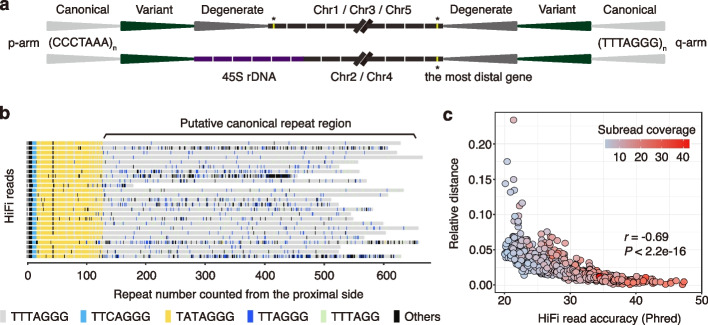
Overview of the telomeric repeat regions in *A. thaliana*. **a** Schematic representation of the different types of telomeric repeats at non-rDNA and rDNA chromosome ends. **b** Alignment of HiFi reads showing the entire telomeric repeat array in chr1q of Pent-46 accession from degenerate, variant to canonical repeats (from left to right). **c** Correlation between relative distance from expected canonical repeat sequence and entire read accuracy

Starting from the centromere-proximal side, the telomeric regions typically start with a stretch of degenerate repeats, followed by variant repeats and finally canonical repeats, all of which were in the same head-to-tail arrangement (Fig. 1a). The most obvious exceptions to this general pattern were chr2p and chr4p ends, where only 11 accessions had variant repeats. Additionally, 30 accessions contained non-telomeric fragments within the repeat arrays, and these are described in detail below.

The arrays of canonical telomeric repeats at the very end were observed to harbor many indels in each read, primarily 1-bp indels, usually replacing TTTAGGG with either TTAGGG or TTTAGG (Fig. 1b; Additional file 1: Fig. S7). By comparing HiFi read accuracy, the number of full-pass subreads, and the relative distance from an ideal sequence that is the entire canonical array for each read, a statistically significant negative correlation was found between relative distance and both read accuracy (*P* < 2.2e − 16, Pearson’s *r* =  − 0.69; Fig. 1c) and subread coverage (*P* < 2.2e − 16, Pearson’s *r* =  − 0.42). Since only indels and no other mutation types were found in this region, the relative distance serves as an indication of indel density. This result suggests that the occurrence of indels is influenced by the read quality. Different from a previous study that interpreted indels supported by a single read as genuine variants [28], we consider these indels to be short homopolymer run errors (e.g., TTT > TT or GGG > GG), a known issue with HiFi reads [34, 39]. Therefore, the region beginning at the last conserved variant repeat until the read end was defined as the homogeneous canonical TTTAGGG repeat region. Because it was deemed to be devoid of consistent variation, this region was not further considered in the remainder of analyses.

### Hypervariable composition of telomeric repeat arrays

**Fig. 2:**
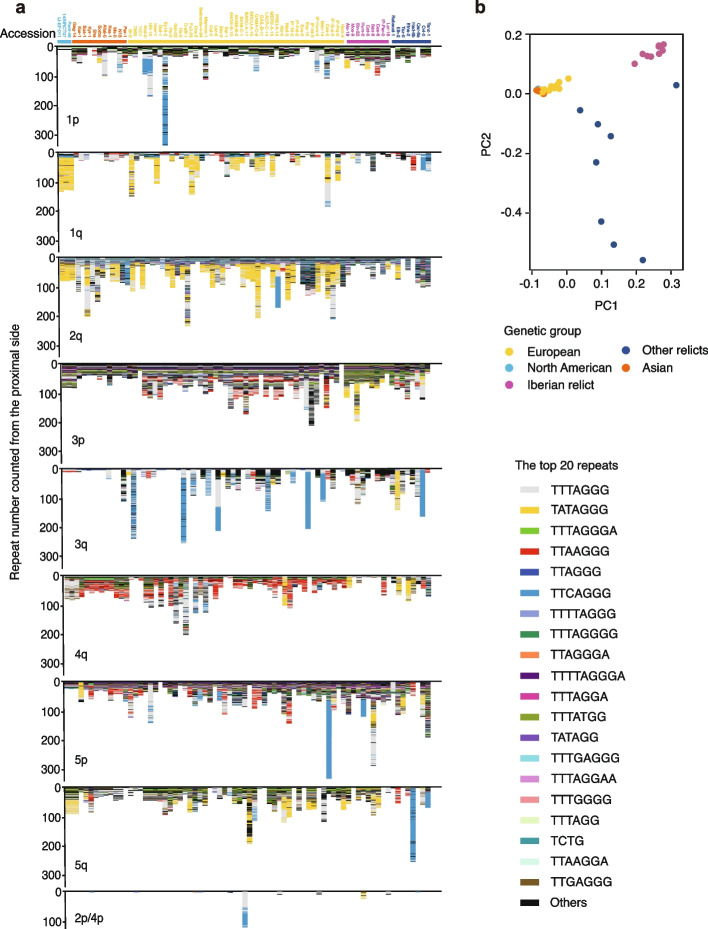
Variation of telomeric sequences in *A. thaliana*. **a** Degenerate and variant telomeric repeat arrays at 10 chromosome ends across all 74 accessions. The top 20 most enriched units are highlighted by different colors. **b** Genetic groups of 74 accessions revealed by principal component analysis

Using the extracted reads, we generated consensus sequences of degenerate and variant telomeric repeats for each chromosome end in the 74 accessions. To obtain a first overview of variation, the 20 most enriched repeat types were visualized (Fig. 2a). Sequences of accessions were ordered according to their membership in genetic groups (Fig. 2b; Additional file 1: Fig. S8). Of the 592 non-rDNA chromosome ends, 562 had variant repeat arrays, with lengths from 6 to 3,384 bp (chr1p of Ey15-2). Of the 148 rDNA ends, only 12 had variant repeat arrays, with lengths from 6 to 658 bp. A total of 462 distinct repeat units, ranging in size from 2 to 17 bp, were identified (Additional file 2: Table S2). The number of new repeats reached saturation with the 69th accession (Additional file 1: Fig. S9). Of the 462 distinct repeat units, 151 (32.7%) occurred only once. The canonical repeat, which was interspersed among arrays of variant repeats, had the highest frequency with 20.7%. It should be noted that the count of distinct repeat types greatly relies on our definition of a unit. For example, the sequence TTTAGGATTAGGG could be considered as being composed of two variant repeats, TTTAGGA and TTAGGG or TTTAGG and ATTAGGG. Therefore, we use the repeat types as a set of markers for studying the overall organization of telomeric sequences and believe that there is no need to excessively focus on the specific sequence content of individual units, especially rare ones.

As an aside, the template sequence of the telomerase RNA, 5′-CUAAACCCU-3′ [40], encoded on chromosome 2, was identical in all 74 accessions (Additional file 2: Table S3).

Although the sequence content of telomeric regions was highly dynamic, there were five main patterns of sequence variation and most variant sequences, 508 of 574, showed more than one of these patterns. The simplest pattern was represented by arrays in which different repeat types occurred only once, such as chr1q of Cat-0 (Fig. 2a). The second pattern most likely resulted from monomer homogenization, such as chr1p of Alo-19, where a single unit, TATAGGG, was repeated consecutively 15 times (Additional file 1: Fig. S10a). The remaining patterns constituted higher-order repeats (HORs) [41]. In the simplest case, such as chr3q of IP-Tri-0, two to four units made up a block that was then repeated multiple times (Additional file 1: Fig. S10b). A more elaborate pattern had multiple monomers (arbitrarily defined as ≥ 5 here) that were repeated several times. For example, in chr2q of IP-Per-0, five distinct units formed a block and were repeated five times, with all five blocks being identical (Additional file 1: Fig. S10c). The final pattern also featured HORs but with mutations distinguishing the individual HORs. For instance, in chr2q of Cvi-0, the HOR array consisted of five units repeated eight times with five of these deviating from the consensus (Additional file 1: Fig. S10d).

When comparing pairs of accessions, the majority of sequence differences between specific chromosome ends fell into three major categories (Fig. 2a). In the first category, sequences were highly similar to each other, as seen in chr5p of 11C1 and HR-10 (Additional file 1: Fig. S11a). In the second group, sequence composition was similar, but accessions were distinguished by the number of HORs, such as chr3p of IP-Tri-0 and IP-Fel-2 (Additional file 1: Fig. S11b). These two categories were mainly observed with pairs from the same genetic group. The third category, sequence divergence, was observed not only in unrelated accessions but also in pairs from exactly the same local population, such as chr1p of Evs-0 and Evs-12 (Additional file 1: Fig. S11c).

Thirty accessions had non-telomeric sequences within the repeat array (Additional file 2: Table S4). Except for seven unclassified sequences ranging in length from 42 to 453 bp, the others could typically be divided into three different types. Firstly, organellar DNA or rDNA insertions. In chr1p, 14 accessions had a 110-bp mitochondrial DNA insertion (Additional file 1: Fig. S12a), which has been reported previously [23], while chr2q of Cvi-0 contained a 102-bp chloroplast DNA insertion. A 5088-bp 45S rDNA sequence was embedded in the telomeric tract in chr2q of Gel-1. In the second type, seven accessions were observed to have non-telomeric fragments that were associated with repeat array duplications. For example, chr2q of four accessions has a 244-bp sequence that forms HORs in combination with their telomeric repeats. The 244-bp fragment is identical in all HOR copies, while the repeat array exhibits a few polymorphisms (Additional file 1: Fig. S12b). The third type was exemplified by chr3q of Hum-2, where the repeat array was interrupted by a 495-bp non-telomeric fragment, which was identical in sequence to a fragment adjacent to the array of variant telomeric repeats of chr5q in the same accession (Additional file 1: Fig. S12c). The distal part of chr3q closely resembled the repeat array of chr5q.

### Repeat number variation between closely related individuals and in somatic tissues

To examine variability in the telomere regions in a more fine-grained manner, two collections of datasets from very closely related individuals were employed. The first collection came from the lineage of North American accessions known as haplogroup-1 (HPG1), which form a clade of natural mutation accumulation lines whose common ancestor lived about 400 years ago [42]. In parallel, three independent sequencing datasets of the Col-0 accession that had been recently published were investigated [30, 31, 43]. This also offered an opportunity to examine intra-dataset variation in more detail. We therefore report not merely the most common repeat array length but present the full data for all HiFi reads.

**Fig. 3:**
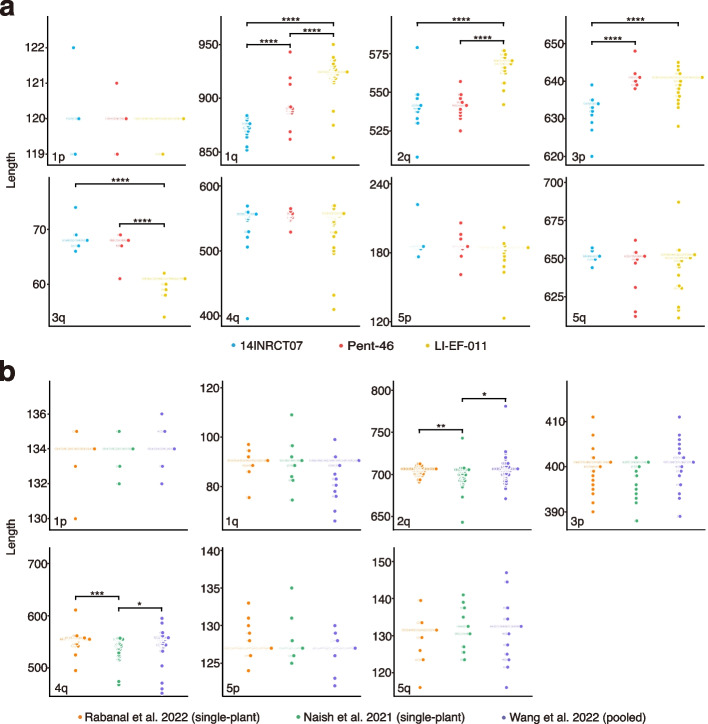
Variation in the lengths of degenerate and variant telomeric repeat regions in sets of three closely related samples. Dots represent individual HiFi reads. Statistically significant differences were determined by a two-tailed *F* test (****P < 0.00001, ***P < 0.0001, **P < 0.001, *P < 0.01). **a** Comparison of the three HPG1 accessions. **b** Comparison of the three Col-0 datasets

Among the three HPG1 accessions, repeat number variation was found, but no major differences were observed in repeat type. Specifically, four of eight non-rDNA chromosome ends were significantly different in lengths of degenerate and variant repeat regions, with medians differing from 7 to 51 bp (Fig. 3a). There was also substantial variation in repeat number among the HiFi reads from a single accession. The greatest one, from 396 to 569 bp, corresponding to approximately 25 repeat units, was observed at chr4q of 14INRCT07(Fig. 3a).

In the Col-0 accession, the array of telomeric repeats of chr3q was found to exclusively consist of canonical repeats, and it was therefore excluded from this analysis. For the remaining seven non-rDNA chromosome ends, there was no difference in variant types. Regarding repeat number variation, at two of seven chromosome ends, one dataset differed significantly in length distribution from the other two datasets, with median differences of 7 bp and 11 bp (Fig. 3b). These two chromosome ends had also the longest repeat arrays. For within-dataset length variation, chr4q was the one with the greatest difference between the shortest and longest arrays of degenerate and variant repeats, at 184 bp, roughly equivalent to 26 repeats (Fig. 3b). While four of seven chromosome ends differed significantly in the degrees of variability among the three Col-0 datasets (Additional file 1: Fig. S13), these differences were not attributable to the pooled-sequencing dataset. Thus, differences in sequencing strategy should not affect our conclusions regarding the 74 diverse datasets we used, which had been generated by a combination of pooled and single-plant sequencing.

### Haplotype structure of telomeric repeat arrays and the adjacent non-coding regions

To facilitate the comparison of haplotypes across the telomeric arrays, we implemented a repeat compression process to mitigate the impact of repeat number variation, which is likely to change more quickly than the overall arrangement and presence of variant repeats (Additional file 1: Fig. S14). The compressed sequences were used to perform a pairwise sequence similarity analysis based on the relative Levenshtein distance (L-distance) [44]. The result confirmed the visual impression from Fig. 2a that there is on average more similarity between the same chromosome end of different accessions than between different chromosome ends (Additional file 1: Fig. S15a; *P* < 2.2e − 308, Wilcoxon test). The result also showed an overall lower relative L-distance within the same genetic group compared to between different genetic groups (Additional file 1: Fig. S15b; *P* < 6.01e − 59, Wilcoxon test).

**Fig. 4:**
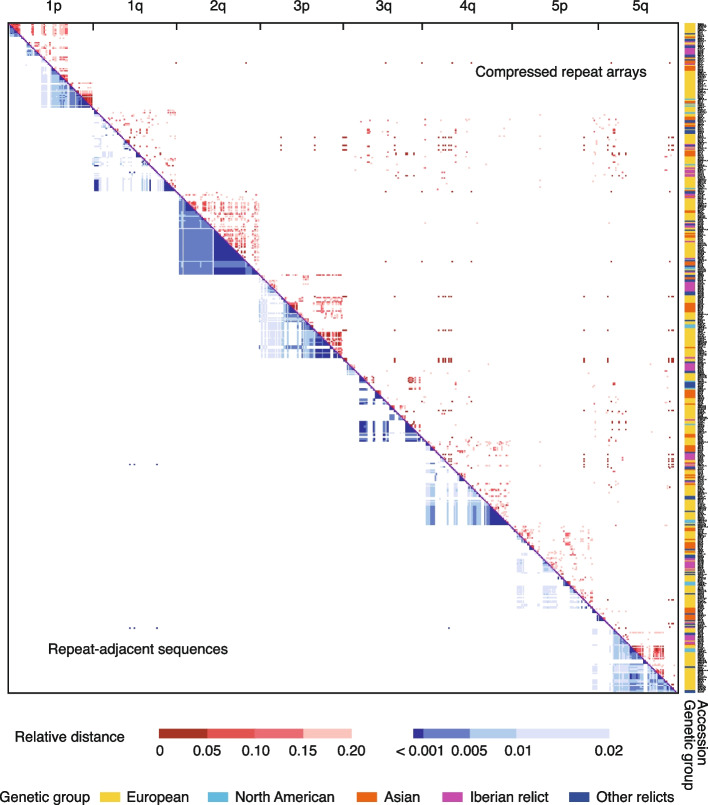
Heatmap of pairwise relative distance of compressed telomeric repeat arrays (upper triangle) and repeat-adjacent sequences (lower triangle). Membership of accessions in different genetic groups is indicated

To examine whether these haplotype patterns extended beyond the telomeric repeat regions, we also looked at their adjacent non-coding regions. Non-coding sequences, which varied in length from zero to 16,542 bp, were defined as the sequence between the most distal gene and the last variant repeat of each chromosome end (Additional file 2: Table S5). Next, neighbor joining (NJ) clustering was conducted based on the multi-sequence alignment of these non-coding regions from each chromosome end. A merged matrix of repeat arrays and non-coding regions was generated, using the accession order from the NJ exercise, to reveal the correlation between the two (Fig. 4). Strong linkage between telomeric repeats and their adjacent non-coding regions were present at both coarse and fine resolution.

In addition to linkage disequilibrium, the matrix provided direct support for our statistical results regarding the chromosome end-specific and genetic group-specific patterns (Fig. 4). Haplotypes from the same chromosome end clustered together, with accessions from the same genetic group typically having similar haplotypes.

## Discussion

Our study provides a base-level view of the patterns of degenerate and variant telomeric repeats at the chromosome ends of 74 geographically diverse accessions of *A. thaliana*. The diverse sampling combined with technical advances provide a population-level view of telomeric sequence, going far beyond previously available anecdotal observations from a few common accessions [8, 17]. The superior length of PacBio HiFi reads supports unambiguous anchoring of the telomeric repeats to each chromosome end. In previous studies, total repeat abundance was reported without linking repeat location to telomeres in general, let alone to individual telomeres [24], or the focus was on only one chromosome end [23]. The superior read length mitigates the challenges arising from having multiple canonical repeats embedded within the variant repeat array (Fig. 2a), which can otherwise be taken as an erroneous indication of the chromosome end [17]. The number of variant patterns detected in our study reached saturation with the 69th accession of the 74 accessions (Additional file 1: Fig. S9). Therefore, we were able to detect not only mutation types of high frequency such as TTCAGGG [8] but also a much broader range of variant patterns that is likely to provide a near-complete inventory of variant types. Of note, our results overturn the previous conclusion that there are no variants at the two chromosome ends that cap the large 45S rDNA repeat arrays [38]. In addition, we newly discovered higher-order repeats (HORs), which have before only been described in other satellite regions of *A. thaliana* such as the centromere [31]. Regarding inter-chromosome similarity of the unique sequence and its subsequent telomeric tract (Additional file 1: Fig. S12c), the only other similar example that we are aware of comes from *Caenorhabditis elegans* [45]. Thus, our work greatly extends our knowledge of telomere-adjacent sequence variation up to the canonical array in this species.

There is ample evidence for local homogenization of telomeric repeats and formation of HORs, as well as repeat number variation in somatic cells and between closely related individuals, all typical characteristics of non-coding minisatellite regions [41, 46]. The obvious scenario is that only the most distal portions of the canonical repeats, at the very ends of the chromosomes, are maintained by telomerase and thus remain uniform [40]. More centromere-proximal portions are maintained by conventional DNA replication and can sustain mutations, becoming first variant repeats and eventually degenerate repeats over time [23]. In this scenario, the variant and degenerate repeats are minisatellite units of about 7 bp, and the extensive patterns of apparent repeat expansion and contraction can be explained by replication slippage and unequal crossing over [47]. Two other forces shaping variant repeats have been considered in previous studies, and our analyses cannot rule out that they play a minor role as well. Variant repeats could in principle be caused by variation in the RNA template. While we detected no sequence differences at the previously reported locus for the canonical RNA template [40, 48], we cannot exclude the existence of other loci that contribute a minor amount of alternative templates [49]. Alternatively, variants could arise during reverse transcription [50], introducing variants into the newly added repeats at the most distal end of the array. Such errors will cause telomere elongation by telomerase to fail, with alternative mechanisms for telomere maintenance eventually taking over.

Our haplotype analysis revealed both chromosome end-specific and genetic group-specific patterns of degenerate and variant telomeric repeat arrays. Accessions sharing the same haplotype are more likely to belong to the same genetic group [28], but they are not necessarily from the same local population [23]. In addition, we demonstrate that linkage disequilibrium between telomeric repeat arrays and more proximal non-coding regions, previously described for single chromosome ends in humans and *A. thaliana* [14, 23, 51], as a common feature at all non-rDNA chromosome ends in *A. thaliana*. The mitochondrial DNA insertion event observed in accessions is a good example for summarizing these patterns in conjunction with the mutational process we propose (Additional file 1: Fig. S12a). The 14 accessions, from different localities, contain a conserved mitochondrial fragment and highly similar repeat-adjacent sequences, but the repeat arrays differ in sequence. A likely scenario is that the mitochondrial fragment was inserted before these 14 chromosome ends diverged [23]. Base substitutions in the telomeric repeat arrays then occurred stochastically in different accessions during repeat amplification.

Our analysis has shown that telomeric repeats experience apparently much higher mutation rates than high-complexity sequences in chromosome arms, especially when it comes to repeat number. Telomeric repeats are therefore potentially helpful when attempting to reconstruct relationships between closely related individuals at high resolution. Information from telomeric repeats might become particularly useful if combined with genome-wide analyses of microsatellite and minisatellite mutations [52]. The substantial intra-individual variation in telomeric repeats also offers opportunities for studying the mechanisms of replication slippage and unequal crossing over of minisatellites [53], given that the entire telomeric repeat arrays can be confidently captured by single HiFi reads.

Our study leaves several open questions for future studies. One challenge will be to accurately assign telomeric reads adjacent to rDNA to specific chromosome ends, which has so far been hampered by a lack of complete assemblies of rDNA arrays across diverse genomes [54]. Second, a few chromosome ends, including chr5p of Cas-0 as the most extreme example, had a large number of consecutive TTCAGGG repeats (Fig. 2a). The functional implication of this observation remains unknown. Lastly, we observed the sharing of the unique sequence across chromosome ends at chr2q and chr5q of Hum-2 (Additional file 1: Fig. S12c). This configuration, not yet reported in *A. thaliana*, has been proposed in a *C. elegans* study and in several reviews as evidence for chromosome healing, which involves a recombination process after a double-strand break [45, 55–57]. Further validation of the mechanism underlying this sequence arrangement in *A. thaliana* is required.

## Conclusions

We provide a comprehensive evaluation of nucleotide sequence polymorphisms of degenerate and variant telomeric repeat arrays at all chromosome ends in a global collection of diverse *Arabidopsis thaliana* accessions. We have greatly improved on the detection of telomeric repeat types, and report sequence arrangements including higher-order repeats and the sharing of unique fragments across chromosome ends, which to our knowledge had not been observed before in *A. thaliana*. The number of degenerate and variant telomeric repeats can vary at germline and somatic levels in otherwise isogenic accessions. Lastly, we reveal chromosome end-specific and genetic group-specific patterns of telomeric repeat haplotypes along with linkage disequilibrium between telomeric repeat arrays and their adjacent non-coding regions. Together, the findings improve our understanding of telomeric sequence diversity in plants.

## Methods

### HiFi-based data collection

Seventy-three HiFi-based assemblies and read sets, representing 71 natural accessions, were obtained from seven public sources. The datasets of 44 accessions were from Wlodzimierz et al. [35]. 11 from Kang et al. [58], 14 from Lian et al. [59], the Kew-1 accession from Christenhusz et al. [60], and three independent Col-0 datasets from Rabanal et al. [43], Wang et al. [30], and Naish et al. [31].

Three HPG1 accessions [61] were sequenced with one SMRT Cell on the Sequel II platform (PacBio). Plant growth [62], DNA extraction from a single plant [35], preparation of a multiplexed sequencing library followed by HiFi sequencing [43], and genome assembly [35] were performed as previously described.

### Principal component analysis

A principal component analysis (PCA) was performed to elucidate the genetic relationship among the 74 accessions. HiFi reads from all accessions were aligned to the Col-0 Community-Consensus (Col-CC) assembly [63] by minimap2 v2.26 [64] with the parameter -ax map-hifi. The output SAM files were converted to BAM format using Samtools v1.10 [65] functions view -Sb, sort and index. Site depth was calculated from the aligned BAM files with mosdepth [66]. Single-nucleotide polymorphisms (SNPs) were identified using DeepVariant v1.6.0 [67]. GVCF files for each individual and each chromosome were merged into five chromosome files with GLnexus v1.4.1 [68]. Sites with depth lower than 5, greater than twice the mean depth, or with a genotype quality lower than 30 were discarded. Bcftools v1.17 [69] was used to filter SNPs with the parameter -m 2 -M 2 -i ‘QUAL > 30 && MAF > 0.01 && F_missing < 0.2’, to merge VCF files and to exclude repetitive regions identified by SRF [70] along with KMC v3.2.1 [71]. PCA was conducted using GCTA v1.94.1 [72] with input BED files generated by PLINK v1.90b7.2 [73].

### Extraction of telomeric sequences

In *A. thaliana*, two out of ten chromosome ends have large 45S rDNA repeat arrays adjacent to the telomeric repeats, causing most assemblies collapse and thus preventing correct mapping of telomeric sequences [38, 54]. Two alternative strategies were employed to extract telomeric sequences, depending on whether the sequence was adjacent to long 45S rDNA sequences.

For the eight non-rDNA chromosome ends, an alignment-based approach was employed. For each sample, HiFi reads were aligned to the corresponding assemblies. Since the repeat-adjacent regions of different chromosome ends, which serve as markers for uniquely anchoring reads, were known to be similar in sequence [27], all-against-all pairwise alignments of the 5 kb sequence adjacent to the telomeric repeats were performed for each chromosome end with BLAST v2.13.0 + [74]. This resulted in a maximum alignment overlap of 3,056 bp (between chr3q and chr4q of Cvi-0). Therefore, only reads containing at least 3.5 kb of repeat-adjacent sequence were extracted with samtools view -hb -L [17, 28, 32]. BAM files were converted to FASTA format using samtools bam2fq and processed with seqtk v1.3 (https://github.com/lh3/seqtk) using option seq -A. For each accession, an all-against-all alignment was performed on the extracted reads using TIPP (https://github.com/Wenfei-Xian/TIPP). The resulting data were used to generate network graphs with R package igraph [75] to verify the accuracy of the read extraction. Potentially chimeric reads and reads containing sequencing errors were excluded after visual inspection. All reads were manually clipped to remove non-repeat sequences, retaining only the telomeric tracts. Since the irregular degenerate repeat content made the boundary between the non-repeat and repeat portions ambiguous, the start of the telomeric repeat array was arbitrarily defined as the first instance of the sequence (T)_x_(M)(G)_y_(M) (M = A or C).

For the chr2p and chr4p ends, which contain large 45S rDNA arrays, reads were directly extracted without help of the corresponding genome assembly. Using minimap2, reads that aligned to the 45S rDNA sequence of Col-0 [43] were identified. Reads with at least three consecutive telomeric repeats were further retained. The 45S rDNA portions of these retained reads were aligned pairwise using BLAST. It resulted in the length of identical 45S rDNA sequences being either less than 4,800 bp or nearly the entire length of the query sequence. Reads with at least 5 kb of 45S rDNA sequences were thus extracted and clustered into two groups, putatively from chr2p and chr4p, per accession based on sequence similarity. Based on a 45S rDNA reference sequence [43], RepeatMasker v4.0.9 [76] was used to mask and exclude the rDNA regions from the reads, leaving only the telomeric repeats for further analysis.

To facilitate downstream analysis, reads with telomeric repeats in the 3′-CCCTAAA-5′ orientation were first reversed to 5′-TTTAGGG-3′ using seqtk with function seq -r, followed by processing with Tandem repeats finder v4.09.1 [77] to identify repeat units [78, 79]. After manual curation, units were arbitrarily defined as beginning from the first T and ending with the last non-T base along the sequence. For example, the sequence TGTTTAGGGTCTGATGGG was split into the units TG TTTAGGG TCTGA TGGG.

### Evaluation of short homopolymer errors

Because at each end of the reads, small indels rather than SNPs, particularly 1-bp deletions, often dominated the consecutive canonical TTTAGGG repeat regions, specifically TTAGGG (with two instead of three Ts) or TTTAGG (with two instead of three Gs), and these occurred at random positions. To determine whether these indels were caused by somatic mutations or sequencing errors [39], the correlation between the likelihood of errors and the occurrence of indels for each read was examined.

The likelihood of error was quantified based upon subread coverage and quality value of the HiFi reads. Samtools view -X followed by an awk command was used to extract the values of two tags, “np” (number of subreads) and “rq” (read quality), per read from the BAM files. To calculate the occurrence of indels, sequences were extracted from the read end until the variant repeat preceding the canonical repeat array. The length of each extracted sequence was divided by seven to obtain an approximate repeat number, and a hill-climbing algorithm was used to find the nearest integer that represented the canonical repeat number in the ideal sequence (Additional file 1: Fig. S16), minimizing the Levenshtein distance (L-distance) between the extracted sequence (the observed string) and the ideal sequence consisting entirely of canonical repeats (the expected string), obtained with the R package stringdist. This minimized distance was further divided by the length of the extracted sequence to determine the relative distance as an indication of indel density. The correlation between the likelihood of errors and the occurrence of indels for each read was plotted using R package ggplot2, and the R function cor was used to calculate the Pearson correlation coefficient.

### Identification of telomeric repeat content

To visualize the degenerate and variant repeat arrays, consensus sequences were generated from two random reads with the median length of repeat array for each accession and each chromosome end. Conserved units between reads were retained, while nonconserved units were marked as “N.” The frequency of occurrence for each unit type was subsequently calculated. The positions of the top 20 enriched unit types were then emphasized with different colors.

In addition, non-repeat sequences that disturbed the repeat arrays were manually extracted. Using BLAST, the sources of these non-repeat sequences were determined with TAIR10 transposon and organellar DNA sequences [80] as well as a library of *A. thaliana* rDNA and centromere sequences [43].

### Identification of telomerase RNA template sequence

In *A. thaliana*, the addition of telomeric repeats is directed by a 9-bases template 3′-UCCCAAAUC-5′ in the telomerase RNA, corresponding to 3′-TCCCAAATC-5′ in the genome [40]. To investigate whether the variants we observed were caused by mutations in the template sequence, all 74 assemblies were searched using BLAST with the sequence of the telomerase RNA locus of Col-0 as a query [48]. Corresponding sequences were extracted using bedtools v2.27.1 [81] with function getfasta and used as input for a multiple sequence alignment with Clustal Omega [82].

### Estimation of telomeric repeat variants in HPG1 accessions

Three HPG1 accessions (14INRCT07, Pent-46, LI-EF-011) were sequenced. To assess the repeat number variation, the length of the sequences containing degenerate and variant repeats was calculated for each read with an awk script. The significance of the difference in length between accessions was evaluated with a two-tailed *F* test using the R function var.test. The length of each read was plotted using ggplot2.

### Estimation of telomeric repeat variants in different Col-0 datasets

Three datasets of the Col-0 accession [30, 31, 43] were compared using the methods described above. The R function var.test was additionally used to assess whether different sequencing strategies (single-plant versus pooled) affected the distribution of repeat number variation of HiFi reads.

### Haplotype structure analysis of the repeat arrays and their adjacent non-coding regions

For telomeric repeat arrays, a repeat compression approach for each sequence was used [83], in order to reduce the complexity arising from repeat number variation. Pairwise L-distances between compressed arrays were calculated to estimate their similarity, and these distances were then divided by the length of the longer sequence in each pair to determine the relative distance. An *F* test was performed to assess whether there were significant differences in the similarity levels when comparing the same and different chromosome ends and comparing the same and different genetic groups.

To identify the more proximal non-coding regions, Liftoff v1.6.3 [84] was used in conjunction with the TAIR10 gene set [80] to annotate the most terminal gene in the eight non-rDNA chromosome ends [85]. Subsequently, the fragment between the most terminal gene and the first telomeric repeat was extracted using bedtools getfasta. Multiple sequence alignment and NJ clustering of non-coding sequences was performed for each end with Clustal Omega, and pairwise relative distances were calculated.

To determine whether there was any correlation between variation in the telomeric repeat arrays and the non-coding regions, the relative distance values for both the repeat and the non-coding regions were merged into a square matrix. The order of accessions for each chromosome end was determined based on the NJ clustering of the non-coding regions.

## Supplementary Information

Additional file 1: Supplementary figures. Fig. S1. Geographic distribution of the 74 *A. thaliana* accessions. Fig. S2. Sequence relationships of telomeric reads from non-rDNA chromosome ends of accessions 1–20. Fig. S3. Sequence relationships of telomeric reads from non-rDNA chromosome ends of accessions 21–40. Fig. S4. Sequence relationships of telomeric reads from non-rDNA chromosome ends of accessions 41–60. Fig. S5. Sequence relationships of telomeric reads from non-rDNA chromosome ends of accessions 61–74. Fig. S6. Schematic illustration of the strategies for extracting telomeric reads. Fig. S7. Sequence tracks showing the entire telomeric repeat arrays in the eight non-rDNA chromosome ends of the three North American accessions. Fig. S8. Zoomed-in view of principal component analysis of European, North American and Asian genetic groups. Fig. S9. Number of new repeat units added with an increase in the number of accessions. Fig. S10. Close-up of four major types of sequence organization in the telomeric repeat arrays. Fig. S11. Close-up view of three categories of telomeric sequence relationships. Fig. S12. Representation of three categories of non-telomeric fragments in telomeric repeat arrays. Fig. S13. Density plot of the length distribution of degenerate and variant repeat regions at seven non-rDNA chromosome ends in three Col-0 datasets. Fig. S14. Schematic representation of the repeat compression process. Fig. S15. Violin plots showing the distribution of pairwise relative distances. Fig. S16. Example of the process for determining the expected string and calculating the L-distance, which represents the occurrence of indels.

Additional file 2: Supplementary tables. Table S1. Sampling details for 74 *Arabidopsis thaliana* accessions. Table S2. Degenerate and variant telomeric repeat arrays for each chromosome end in each accession. Table S3. Sequence of the template in telomerase RNA for each accession. Table S4. Non-telomeric sequences within telomeric repeat arrays. Table S5. Annotation of the most distal gene at each chromosome end for each accession.

Additional file 3: Review history.

## Data Availability

Publicly available datasets are from EBI ENA (project numbers PRJEB55353, PRJEB55632, PRJEB50694, PRJEB51511, PRJEB62038) [86–90], and GSA (project numbers PRJCA012695 and PRJCA005809) [91, 92]. Data for three HPG1 accessions generated in this study are available from ENA (project number PRJEB75768) [93]. The custom workflow and scripts are available in Zenodo (10.5281/zenodo.13323746) [94] and a GitHub repository (https://github.com/Yue-qi-Tao/Telomeric-diversity) [95] under MIT license.
