# Supplementary material for: Atlas of telomeric repeat diversity in Arabidopsis thaliana
Source: Genome Biol. 2024 Sep 16;25:244. doi: 10.1186/s13059-024-03388-3 (PMC11406999; doi:10.1186/s13059-024-03388-3)
Supplement: Supplementary file 1 — Additional file 1: Supplementary figures. Fig. S1. Geographic distribution of the 74 A. thaliana accessions. Fig. S2. Sequence relationships of telomeric reads from non-rDNA chromosome ends of accessions 1–20. Fig. S3. Sequence relationships of telomeric reads from non-rDNA chromosome ends of accessions 21–40. Fig. S4. Sequence relationships of telomeric reads from non-rDNA chromosome ends of accessions 41–60. Fig. S5. Sequence relationships of telomeric reads from non-rDNA chromosome ends of accessions 61–74. Fig. S6. Schematic illustration of the strategies for extracting telomeric reads. Fig. S7. Sequence tracks showing the entire telomeric repeat arrays in the eight non-rDNA chromosome ends of the three North American accessions. Fig. S8. Zoomed-in view of principal component analysis of European, North American and Asian genetic groups. Fig. S9. Number of new repeat units added with an increase in the number of accessions. Fig. S10. Close-up of four major types of sequence organization in the telomeric repeat arrays. Fig. S11. Close-up view of three categories of telomeric sequence relationships. Fig. S12. Representation of three categories of non-telomeric fragments in telomeric repeat arrays. Fig. S13. Density plot of the length distribution of degenerate and variant repeat regions at seven non-rDNA chromosome ends in three Col-0 datasets. Fig. S14. Schematic representation of the repeat compression process. Fig. S15. Violin plots showing the distribution of pairwise relative distances. Fig. S16. Example of the process for determining the expected string and calculating the L-distance, which represents the occurrence of indels. [file 13059_2024_3388_MOESM1_ESM.docx]

# SUPPLEMENTARY FIGURES

**Fig. S1.** Geographic distribution of the 74 *A. thaliana* accessions. Genetic groups are indicated by colors.

**Fig. S2.** Sequence relationships of telomeric reads from non-rDNA chromosome ends of accessions 1-20. Chromosome ends are indicated by colors.

**Fig. S3.** Sequence relationships of telomeric reads from non-rDNA chromosome ends of accessions 21-40. Chromosome ends are indicated by colors.

**Fig. S4.** Sequence relationships of telomeric reads from non-rDNA chromosome ends of accessions 41-60. Chromosome ends are indicated by colors.

**Fig. S5.** Sequence relationships of telomeric reads from non-rDNA chromosome ends of accessions 61-74. Chromosome ends are indicated by colors.

**Fig. S6.** Schematic illustration of the strategies for extracting telomeric reads. **a** Strategy for the eight non-rDNA chromosome ends. Telomeric reads that contained at least 3.5-kb repeat-adjacent sequences from the relevant genome assembly were extracted. **b** Strategy for the two rDNA-binding chromosome ends. Reads containing 45S rDNA sequence and more than three consecutive telomeric repeats were extracted without the help of genome assemblies and clustered into two groups based on sequence similarity of the 45S rDNA region.

**Fig. S7.** Sequence tracks showing the entire telomeric repeat arrays in the eight non-rDNA chromosome ends of the three North American accessions from degenerate, variant to canonical repeats (from left to right). Two reads were randomly selected per accession per end. Putative canonical repeat regions are indicated.

**Fig. S8.** Close-up view of principal component analysis of European, North American and Asian genetic groups.

**Fig. S9.** Number of new repeat units added with an increase in the number of accessions.

**Fig. S10.** Close-up view of four major types of sequence organization in the telomeric repeat arrays. Color code corresponds to that of Figure 2. **a** An example of monomer homogenization. In this case, a single unit is repeated 15 times. **b** An example of simple block expansion. In this case, two units form a block, and the block is repeated ten times. **c** An example of expansion of identical higher-order repeats (HORs). A HOR consisting of five distinct units is repeated five times. **d** An example of HORs with small sequence differences. Numbers indicate different HORs.

**Fig. S11.** Close-up view of three categories of telomeric sequence relationships. **a** Telomeric sequences in chr5p of 11C1 and HR-10 are similar. **b** Telomeric sequence in chr3p of IP-Fel-2 has one more copy of a higher-order repeat than the sequence in chr3p of IP-Tri-0. **c** Telomeric sequences in chr1p of Evs-0 and Evs-12 are dissimilar.

**Fig. S12.** Representation of three categories of non-telomeric fragments in telomeric repeat arrays. **a** A 110-bp mitochondrial DNA insertion in 14 accessions. **b** Higher-order repeats in four accessions include a 244-bp non-telomeric fragment. **c** A 495-bp unique sequence in chr3q of Hum-2 is also found in chr5q of Hum-2.

**Fig. S13.** Density plot of the length distribution of degenerate and variant repeat regions at seven non-rDNA chromosome ends in three Col-0 datasets. Statistically significant differences in the deviation degree are indicated (****P<0.00001, ***P<0.0001, **P<0.001, *P<0.01, *F* test).

**Fig. S14.** Schematic representation of the repeat compression process.

**Fig. S15.** Violin plots showing the distribution of pairwise relative distances. Statistically significant differences between accessions are indicated (****P<0.00001, two-tailed F test). **a** Comparison of values within and between chromosome ends. **b** Comparison of values within and between genetic groups.

**Fig. S16.** Example of the process for determining the expected string and calculating the L-distance, which represents the occurrence of indels. Blue blocks indicate variant repeats, while gray blocks indicate canonical repeats. ‘S’ indicates ‘string’, ‘n’ should be an integer.
