# Supplementary material for: Atlas of telomeric repeat diversity in Arabidopsis thaliana
Source: Genome Biol. 2024 Sep 16;25:244. doi: 10.1186/s13059-024-03388-3 (PMC11406999; doi:10.1186/s13059-024-03388-3)
Supplement: Supplementary file 3 — Additional file 3: Review history. [file 13059_2024_3388_MOESM3_ESM.docx]

Review History

**First round of review**

**Reviewer 1**

**Were you able to assess all statistics in the manuscript, including the appropriateness of statistical tests used?**

Yes, and I have assessed the statistics in my report.

**Comments to author:**

Tao et al. have investigated the patterns of degenerate and variant telomeric repeats via public HiFi reads from 49 Arabidopsis thaliana accessions. The authors conducted an extensive analysis of telomeric repeats to try to explore the evolutionary mechanisms diversity of telomeric repeats. The topic is infesting and would bring new insights to the current understanding on the structure and evolution of chromosomes' telomeric region. However, this manuscript primarily focuses on elucidating the differences in the composition and variation of telomere sequences between different accessions of A. thaliana, and the insights provided into the mechanism of telomere evolution or its potential biological significance is limited. Especially, the main conclusion stated in abstract is not well supported by evidences in current manuscript. And there are some concerns regarding the results and methods for the improvement of this manuscript.

Major concerns

(1) The study lacks the necessary analyses of chromosomal recombination events. It is important to note that the conclusion stated in the abstract, 'The identification of segmental duplications and at least one inter-chromosomal recombination event suggests a plausible history of how intact telomeres can be maintained in a telomerase-independent manner,' is not well-supported in current manuscript.

(2) For the extraction of telomeric sequences, the first step involved aligning the HiFi reads to the corresponding assemblies. Considering that highly repetitive sequences of telomeres allow very few T2T assemblies of A. thaliana genomes. It is unclear whether the extraction of telomeric sequences was influenced by using T2T or non-T2T assembly. Additionally, were it able to determine if the telomeric sequences fully assembled or extracted considering the complexity structure?

The second step involved performing all-against-all pairwise alignments of the 5 kb sequence adjacent to the telomeric repeats with BLAST, which resulted in a maximum alignment overlap of 3 kb. However, only reads containing at least 4 kb was extracted. It is unclear how were the threshold determined, and whether alternative thresholds would affect the interpretation.

(3) The authors investigated the composition of telomeric repeat arrays of different accessions of A. thaliana. Previous studies have suggested that the length of telomeric sequences can be influenced by genetic and environmental factors. However, the authors only used 49 accessions of A. thaliana, most of which were of European origin. It is uncertain whether these materials are representative enough of the global A. thaliana population. Furthermore, it is unclear whether the number of telomeric repeat arrays identified using these materials has reached saturation.

(4) The conclusion states that the proportion of indels is related to read accuracy. However, in Figure1C and its legend, relative distance and edit distance are used. It is unclear whether the calculation of relative distances would be related to the proportion of indels. Additionally, it is unclear whether other variants, such as SNPs, are taken into account in calculating the distances. The authors need to make it clarified.

(5) In Figure2, a large number of TTCAGGG type repeats are present at the ends of the chromosomes in some of the accessions, making the length of the degenerate and variant repeat region significantly longer in these accessions. The authors explained this observation as is caused by ALT. However, the relevant literature cited in this manuscript, the mechanism of ALT is found in cancer cells. I am curious that if there is any relevant discovery in plant? Or else, it is too weak to make the current inference.

(6) For Figure 5, it is relatively easy to understand that the strong linkage between telomeric repeats and their adjacent non-coding regions within the same chromosome. However, it is unclear why strong linkage disequilibrium can be detected between 1q, 3q, and 4q, but not between 1p and 3p?

Minor concerns

All figures in the manuscript are not well presented and needed to be improved, such as making the colors and labels easier to understand. For example, wheat the ruler of the y-axis in Figure 2? Figure 1B is hard to understand, and what is the range of x-axis?

The conclusion "the majority of sequence differences between specific chromosome ends fell into three major groups (Fig. 2)" in the paper (Lines 38-39) cannot be found in the Figure2.

Figure S2: Please make the Schematic illustration of the strategies for extracting telomeric reads more clearly.

Fig1B: Please label all similar pictures with a specific length.

Figure 4, statistic method for significance analysis should be clearly provided.

Line 37 (page 3): The supplemental table should be labeled with exactly which three accessions were additionally sequenced in this study.

Line 45 (page 3): "chr4ps" should be "chr4p".

Line 37 (page 7): The type of repeat that the color represents should be specified.

Line 7 (page 17): The reads for the three HPG1 accessions need an accession number.

**Reviewer 2**

**Were you able to assess all statistics in the manuscript, including the appropriateness of statistical tests used?**

No, I do not feel adequately qualified to assess the statistics.

**Comments to author:**

The study is detailed in its description of the nucleotide variations at telomeres using long-read sequencing in 49 geographically diverse accessions of Arabidopsis thaliana and reports significant telomeric repeat variations. Telomeric repeat sequences and length are known to demonstrate variability within a species. Vozárová et al, (2022, Plant J) showed a high diversity of repeats in Arabidopsis, identifying canonical Arabidopsis-type telomeric TTTAGGG repeats at chromosome ends and at least four major telomeric variants at pericentromeric regions. Additionally, Belyayev et al. (2023, BMC Genomics) recently reported telomere variability in natural plant populations. The authors have not specified how the variation observed in this study compares to that observed in these prior studies. Moreover, these and many other studies on telomere diversity are not cited and the extent of advance over previous studies remains unclear. Furthermore, I have the following concerns:

1.The authors describe that the telomeric repeat variations they have found are higher than expected but have not thoroughly described previous findings on telomeric variations in Arabidopsis and other plants. It is important to describe the expected telomeric repeat variation based on previous studies and how their findings differ from these expectations.

2. What is the sequencing depth of PacBio datasets utilized? The sequencing depth can influence the repeat number variations reported in different accessions. Is the repeat number variation normalized for the sequence depth?

3. It is still possible that a large fraction of telomeric repeats is homogeneous, and only a small fraction present near the transition zones contains high variations. What is the total length of telomeric arrays in Arabidopsis? Is it possible that the authors are capturing only telomeric repeat arrays adjacent to the anchoring reads toward chromosomal arms? A bottom-up approach is needed to determine the total number of telomeric repeats. Next, the fraction of telomeric repeats mapping to the anchoring regions should be determined based on this total number of repeats. This will provide a comprehensive view of canonical and variant telomeric repeat fractions.

4. The work does not explore the functional consequences of the telomeric variation at the chromatin level or telomeric maintenance perspective in any capacity.

4. The authors describe non-repeat sequences within telomeric repeat arrays. However, all three types of these so-called non-repeat arrays are actually repeats—rDNA, higher-order repeats, and repeat variants. Therefore, referring to these sequences as non-telomeric repeats or repeat variants would be more appropriate.

Minor comment: What is the significance of the transition zone with degenerate repeats?

**Authors Response**

**Point-by-point responses to the reviewers’ comments:**

**Reviewer #1:**

Tao et al. have investigated the patterns of degenerate and variant telomeric repeats via public HiFi reads from 49 Arabidopsis thaliana accessions. The authors conducted an extensive analysis of telomeric repeats to try to explore the evolutionary mechanisms diversity of telomeric repeats. The topic is infesting and would bring new insights to the current understanding on the structure and evolution of chromosomes' telomeric region. However, this manuscript primarily focuses on elucidating the differences in the composition and variation of telomere sequences between different accessions of A. thaliana, and the insights provided into the mechanism of telomere evolution or its potential biological significance is limited. Especially, the main conclusion stated in abstract is not well supported by evidences in current manuscript. And there are some concerns regarding the results and methods for the improvement of this manuscript.

*Reply: We appreciate the reviewer’s constructive criticism and are grateful for their time and help. We list each of the specific comments and address each of them below.*

Major concerns

The study lacks the necessary analyses of chromosomal recombination events. It is important to note that the conclusion stated in the abstract, 'The identification of segmental duplications and at least one inter-chromosomal recombination event suggests a plausible history of how intact telomeres can be maintained in a telomerase-independent manner,' is not well-supported in current manuscript.

*Reply: Thanks for your valuable suggestion. We agree with your point that the observations on segmental duplication and inter-chromosomal similarity on their own are not sufficient to propose a telomerase-independent mechanism. Therefore, we have deleted the sentences related to the telomerase-independent mechanism from the abstract and throughout the manuscript. The revised Results section of the abstract reads as follows*

*RESULTS: We have comprehensively characterized the sequence variation immediately adjacent to the canonical telomeric repeat arrays at the very ends of chromosomes in 74 genetically diverse Arabidopsis thaliana accessions. We first describe several types of distinct telomeric repeat units and then identify evolutionary processes such as local homogenization and higher-order repeat formation that shape diversity of chromosome ends. By comparing largely isogenic samples, we also determine repeat number variation of the degenerate and variant telomeric repeat array at both the germline and somatic levels. Finally, the analysis of haplotype structure uncovers chromosome end-specific patterns in variant telomeric repeat distribution, and linkage with the more proximal non-coding region.*

*At the same time, we list the observation of the unique non-telomeric fragment between chromosomes in the last paragraph of the Discussion section as an open question for further investigation. We added*

*Lastly, we described the sharing of unique sequence across chromosome ends at chr2q and 5q of Hum-2 (Fig. S6c). This observed configuration, not yet reported in A. thaliana, has been proposed in a C. elegans study and in several reviews as evidence of chromosome healing, which involves a recombination process after a double-strand break [45,55,56,57]. Further validation of the mechanism underlying this sequence arrangement in A. thaliana is required.*

(2) For the extraction of telomeric sequences, the first step involved aligning the HiFi reads to the corresponding assemblies. Considering that highly repetitive sequences of telomeres allow very few T2T assemblies of A. thaliana genomes. It is unclear whether the extraction of telomeric sequences was influenced by using T2T or non-T2T assembly. Additionally, were it able to determine if the telomeric sequences fully assembled or extracted considering the complexity structure?

The second step involved performing all-against-all pairwise alignments of the 5 kb sequence adjacent to the telomeric repeats with BLAST, which resulted in a maximum alignment overlap of 3 kb. However, only reads containing at least 4 kb was extracted. It is unclear how were the threshold determined, and whether alternative thresholds would affect the interpretation.

*Reply: Thank you for your questions!*

*Standard assembly practices with hifiasm (Cheng et al. 2021) work very well in A. thaliana. All eight non-rDNA chromosome ends of the 74 accessions are represented in the assemblies, each with several canonical telomeric repeats at the very end. Therefore, we consider the assemblies as robust and differences between assemblies having no negative impact on our study.*

*We are confident that all reads we extracted can be clearly assigned to specific chromosome ends. This is because in A. thaliana, the sequence structure of the repeat-adjacent region, which we used for anchoring, is quite simple, and sequence similarity across accessions is sufficient for differentiation between chromosome ends. As we explained in the Methods section*

*Since the repeat-adjacent regions of different chromosome ends, which serve as markers for uniquely anchoring reads, were known to be similar in sequence, all-against-all pairwise alignments of the 5 kb sequence adjacent to the telomeric repeats were performed for each chromosome end with BLAST v2.13.0+ . This resulted in a maximum alignment overlap of 3,056 bp kb. Therefore, only reads containing at least 3.5 kb of repeat-adjacent sequence were extracted with samtools view -hb -L.*

*To provide direct evidence for the above two assertions and to demonstrate the robustness of our method, we performed an additional analysis to cluster reads for each accession based on their pairwise sequence similarity in the anchor region. The results show that in every accession, the reads clearly cluster into eight distinct groups (Reviewer Figure 1; Reviewer Figure 2; Reviewer Figure 3; Reviewer Figure 4).*

***Reviewer Figure 1.*** *Sequence relationships of telomeric reads from non-rDNA chromosome ends of accessions 1-20. Chromosome ends are indicated by colors.*

***Reviewer Figure 2.*** *Sequence relationships of telomeric reads from non-rDNA chromosome ends of accessions 21-40. Chromosome ends are indicated by colors.*

***Reviewer Figure 3.*** *Sequence relationships of telomeric reads from non-rDNA chromosome ends of accessions 41-60. Chromosome ends are indicated by colors.*

***Reviewer Figure 4.*** *Sequence relationships of telomeric reads from non-rDNA chromosome ends of accessions 61-74. Chromosome ends are indicated by colors.*

*The underlying logic of parameter setting is to ensure that each read contains a sufficient amount of unique sequence for anchoring. Since 3,056 bp is the maximum overlap length, theoretically, we only need to choose a threshold of 3,056 bp. Conventionally, we choose an integer or half-integer. In the original version, we chose the nearest integer as the parameter. We have now modified the parameter to be more precise, using the nearest half-integer. The revised sentence is*

*This resulted in a maximum alignment overlap of 3,056 bp (between chr3q and chr4q of Cvi-0). Therefore, only reads containing at least 3.5 kb of repeat-adjacent sequence were extracted with samtools view -hb -L*

*The only consequence of changing the parameter from 4 kb to 3.5 kb is that 247 of 592 chromosome ends lose between 1 to 9 candidate reads (Reviewer Figure 5). Since having two reads is already sufficient to generate a consensus sequence, this change will not affect further interpretation. To make our strategy clearer, we have modified the sentence in the Methods section as follows*

*To visualize the degenerate and variant repeat arrays, consensus sequences were generated from two random reads with the median length of the repeat array for each accession and each chromosome end.*

***Reviewer Figure 5.*** *Fraction of the number of coverage changes in candidate reads for all non-rDNA chromosome ends across all accessions.*

(3) The authors investigated the composition of telomeric repeat arrays of different accessions of A. thaliana. Previous studies have suggested that the length of telomeric sequences can be influenced by genetic and environmental factors. However, the authors only used 49 accessions of A. thaliana, most of which were of European origin. It is uncertain whether these materials are representative enough of the global A. thaliana population. Furthermore, it is unclear whether the number of telomeric repeat arrays identified using these materials has reached saturation.

*Reply: Following the reviewer’s comment, we have increased the number of accessions to 74 (Fig. S1; Table S1), and conducted a principal component analysis to better illustrate their genetic groups (Fig. 2b). We have also added the following explanation in the Results*

*Sixty-six accessions were grouped into four main genetic clusters, with 43 non-relict accessions from Europe, 11 from Asia, 9 Iberian relicts, and three from North America. Eight further accessions were from different relict groups.*

*And the Methods*

*Seventy-three HiFi-based assemblies and read sets, representing 71 natural accessions, were obtained from seven public sources. The datasets of 44 accessions were from Wlodzimierz et al. [35]. 11 from Kang et al. [58], 14 from Lian et al. [59], the Kew-1 accession from Christenhusz et al. [60], and three independent Col-0 datasets from Rabanal et al. [43], Wang et al. [30], and Naish et al. [31].*

***Principal component analysis***

*A principal component analysis (PCA) was performed to elucidate the genetic relationship among the 74 accessions. HiFi reads from all accessions were aligned to the Col-0 Community-Consensus (Col-CC) assembly (NCBI BioProject* [*PRJNA915353*](https://www.ncbi.nlm.nih.gov/bioproject/915353)*) by minimap2 v2.26 [63] with the parameter -ax map-hifi. The output SAM files were converted to BAM format using Samtools v1.10 [64] functions view -Sb, sort and index. Site depth was calculated from the aligned BAM files with mosdepth [65]. Single-nucleotide polymorphisms (SNPs) were identified using DeepVariant v1.6.0 [66]. GVCF files for each individual and each chromosome were merged into five chromosome files with GLnexus v1.4.1 [67]. Sites with depth lower than 5, greater than twice the mean depth, or with a genotype quality lower than 30 were discarded. Bcftools v1.17 [68] was used to filter SNPs with the parameter -m 2 -M 2 -i 'QUAL>30 && MAF>0.01 && F_missing <0.2' , to merge VCF files and to exclude repetitive regions identified by SRF [69] along with KMC v3.2.1 [70]. PCA was conducted using GCTA v1.94.1 [71] with input BED files generated by PLINK v1.90b7.2 [72].*

*We have also reanalyzed and updated the results on the variation of telomeric repeat patterns for all 74 accessions (Fig. 2a; Fig. S5; Fig. S6; Fig. S7). The revised paragraph in the Results section is as follows:*

*Using the extracted reads, we generated consensus sequences of degenerate and variant telomeric repeats for each chromosome end in the 74 accessions. To obtain a first overview of variation, the 20 most enriched repeat types were visualized (Fig. 2A), and sequences of accessions were displayed according to their genetic classification (Fig. 2B). Of the 592 non-rDNA chromosome ends, 562 had variant repeat arrays, with lengths from 6 to 3,384 bp (chr1p of Ey15-2). Of the 148 rDNA ends, only 12 had variant repeat arrays, with lengths from 6 to 658 bp. A total of 462 distinct repeat units, ranging in size from 2 to 17 bp, were identified (Table S2). The number of new repeats reached saturation with the 69^th^ accession (Fig. S4). Of the 462 distinct repeat units, 151 (32.7%) occurred only once. The canonical repeat, which was interspersed among arrays of variant repeats, had the highest frequency with 20.7%.*

*Thirty accessions had non-telomeric sequences within the repeat array (Table S4). Except for seven unclassified sequences ranging in length from 42 to 453 bp, the others could typically be divided into three different types. Firstly, organellar DNA or rDNA insertions. In chr1p, 14 accessions had a 110-bp mitochondrial DNA insertion (Fig. S7a), which has been reported previously [23], while chr2q of Cvi-0 contained a 102-bp chloroplast DNA insertion. A 5,088-bp 45S rDNA sequence was embedded in the telomeric tract in chr2q of Gel-1. In the second type, seven accessions were observed to have non-telomeric fragments that were associated with repeat array duplications. For example, chr2q of four accessions has a 244-bp sequence that forms HORs in combination with their telomeric repeats. The 244-bp fragment is identical in all HOR copies, while the repeat array exhibits a few polymorphisms (Fig. S7b). The third type was exemplified by chr3q of Hum-2, where the repeat array was interrupted by a 495-bp non-telomeric fragment, which was identical in sequence to a fragment adjacent to the array of variant telomeric repeats of chr5q in the same accession (Fig. S7c). The distal part of chr3q closely resembled the repeat array of chr5q.*

*As mentioned in the text above, for these 74 accessions, saturation of unique repeats was reached by the 69^th^ accession. The last five accessions do not exhibit any new repeat types (Reviewer Figure 6).*

***Reviewer Figure 6.*** *Number of new repeat units added with an increase in the number of accessions.*

(4) The conclusion states that the proportion of indels is related to read accuracy. However, in Figure1C and its legend, relative distance and edit distance are used. It is unclear whether the calculation of relative distances would be related to the proportion of indels. Additionally, it is unclear whether other variants, such as SNPs, are taken into account in calculating the distances. The authors need to make it clarified.

*Reply: Thanks for pointing this out! The Levenshtein distance (Levenshtein 1966) we used counts any base difference between two strings, regardless of whether it is a substitution or an indel. However, we did not find any substitution-type differences in the putative canonical repeat array; only indels were observed. Therefore, the relative distance is exclusively caused by indels, and the distance value indicates the occurrence of indels. The more indels, the higher the value. To clarify this, we have modified sentences in the Results*

*The arrays of canonical telomeric repeats at the very end were observed to harbor many indels in each read, primarily 1-bp indels, usually replacing TTTAGGG with either TTAGGG or TTTAGG (Fig. 1b; Fig. S3). By analyzing the correlation between HiFi read accuracy, the number of full-pass subreads, and the relative distance from an ideal sequence that is the entire canonical array for each read, a statistically significant negative correlation was found between relative distance and both read accuracy (P < 2.2e-16, Pearson’s r = -0.69; Fig. 1c) and subread coverage (P < 2.2e-16, Pearson’s r = -0.42). Since only indels and no other mutation types were found in this region, the relative distance serves as an indication of indel density.*

*and Methods*

*Because at each end of the reads, small indels rather than SNPs, particularly 1-bp deletions, often dominated the consecutive canonical TTTAGGG repeat regions, specifically TTAGGG (with two instead of three Ts) or TTTAGG (with two instead of three Gs), and these occurred at random positions.*

(5) In Figure2, a large number of TTCAGGG type repeats are present at the ends of the chromosomes in some of the accessions, making the length of the degenerate and variant repeat region significantly longer in these accessions. The authors explained this observation as is caused by ALT. However, the relevant literature cited in this manuscript, the mechanism of ALT is found in cancer cells. I am curious that if there is any relevant discovery in plant? Or else, it is too weak to make the current inference.

*Reply: Thanks for catching this and bringing it to our attention! We did not find studies on plants. Therefore, we have removed the sentence in the Results section and the entire paragraph discussing ALT and telomerase-independent mechanisms from the Discussion section.*

Instead, we mention the observation of a long TTCAGGG array as an open question in the last paragraph of the Discussion section as follows.

*Second, a few chromosome ends, including chr5p of Cas-0 as the most extreme example, had a large number of consecutive TTCAGGG repeats (Fig.2a). The functional implication of this observation remains unknown.*

(6) For Figure 5, it is relatively easy to understand that the strong linkage between telomeric repeats and their adjacent non-coding regions within the same chromosome. However, it is unclear why strong linkage disequilibrium can be detected between 1q, 3q, and 4q, but not between 1p and 3p?

*Reply: We apologize for the unclear visualization. The confusion arose because we initially used absolute Levenshtein distance to measure pairwise sequence similarity. We realized that the absolute distance can be affected by the length of the sequences. Specifically, sequences from chr1q, chr3q and chr4q include pairs where both sequences are relatively short, resulting in smaller absolute distance values.*

*To address this, we have provided an updated Figure 4 (previously Figure 5) with relative distance. This measure normalizes the original absolute distance by dividing it by the longer length in each pair. In the modified figure, strong linkage between the telomeric repeat array and their adjacent non-coding region at each chromosome end is clearly apparent, which is the primary purpose of this figure.*

Minor concerns

All figures in the manuscript are not well presented and needed to be improved, such as making the colors and labels easier to understand. For example, wheat the ruler of the y-axis in Figure 2? Figure 1B is hard to understand, and what is the range of x-axis?

*Reply: Thank you for your suggestions! We have added the y-axis as the repeat number count from the proximal side in Figure 2a.*

*In Figure 1b, we have added the x-axis as repeat number count from the proximal side. Additionally, we have changed the chromosome end shown from chr3q to chr1q which features a longer degenerate and variant repeat region. We have also indicated that the bars represent HiFi reads, making the figure easier to understand.*

The conclusion "the majority of sequence differences between specific chromosome ends fell into three major groups (Fig. 2)" in the paper (Lines 38-39) cannot be found in the Figure2.

*Reply: Thank you for pointing this out. We apologize for the confusion. In the text, we meant to refer to the three categories we explain in this paragraph. For further clarification, we have added in Fig. S6 close-up views of the examples we used for the three categories. We have also modified the description as follows*

*When comparing pairs of accessions, the majority of sequence differences between specific chromosome ends fell into three major categories.*

Figure S2: Please make the Schematic illustration of the strategies for extracting telomeric reads more clearly.

*Reply: We have added annotations to each sequence fragment to make it clearer.*

Fig1B: Please label all similar pictures with a specific length.

*Reply: We have added the x-axis as the repeat number count from the proximal side in Fig. 1b and Fig. S3.*

Figure 4, statistic method for significance analysis should be clearly provided.

*Reply: We have added the statistic method to the figure legend as Statistically significant differences shown were determined by two-tailed F test.*

Line 37 (page 3): The supplemental table should be labeled with exactly which three accessions were additionally sequenced in this study.

*Reply: We have marked the three accessions in Table S1 as Generated in this study.*

Line 45 (page 3): "chr4ps" should be "chr4p".

*Reply: Thank you for noticing this. We have corrected it.*

Line 37 (page 7): The type of repeat that the color represents should be specified.

*Reply: We have incorporated the meaning of different fragments and colors into the figure (now in Fig. S7).*

Line 7 (page 17): The reads for the three HPG1 accessions need an accession number.

*Reply: We have added the sentence Data for three HPG1 accessions generated in this study are available from ENA (project number PRJEB75768).*

**Reviewer #2:**

The study is detailed in its description of the nucleotide variations at telomeres using long-read sequencing in 49 geographically diverse accessions of Arabidopsis thaliana and reports significant telomeric repeat variations. Telomeric repeat sequences and length are known to demonstrate variability within a species. Vozárová et al, (2022, Plant J) showed a high diversity of repeats in Arabidopsis, identifying canonical Arabidopsis-type telomeric TTTAGGG repeats at chromosome ends and at least four major telomeric variants at pericentromeric regions. Additionally, Belyayev et al. (2023, BMC Genomics) recently reported telomere variability in natural plant populations. The authors have not specified how the variation observed in this study compares to that observed in these prior studies. Moreover, these and many other studies on telomere diversity are not cited and the extent of advance over previous studies remains unclear.

*Reply: Thank you for your time to review our manuscript. Your thoughtful and constructive review is much appreciated.*

*Following the reviewer’s suggestion, we have now cited Vozárová et al, (2022, Plant J) in the introduction as the authors reported the existence of degenerate repeats, and Stephens and Kocher (2024, BMC Bioinformatics), which reported variant repeat diversity among chromosome ends. We have also cited Belyayev et al. (2023, BMC Genomics) in the Methods section for their use of tandem repeat finder as a strategy, along with Lyčka et al. (2024, Nucleic Acids Research).*

*We have rewritten the first paragraph of the Discussion section to explain how our study improves upon previous studies. The revised paragraph is attached below as response to the reviewer’s first major concern.*

Furthermore, I have the following concerns:

The authors describe that the telomeric repeat variations they have found are higher than expected but have not thoroughly described previous findings on telomeric variations in Arabidopsis and other plants. It is important to describe the expected telomeric repeat variation based on previous studies and how their findings differ from these expectations.

*Reply: Thank you for valuable suggestions. To better explain the advance of our study, we have rewritten the first paragraph of the Discussion section as follows*

*Our study provides a base-level view of the patterns of degenerate and variant telomeric repeats at the chromosome ends of 74 geographically diverse accessions of A. thaliana. The diverse sampling combined with technical advances provide a population-level view of telomeric sequence, going far beyond previously available anecdotal observations from a few common accessions [8,17]. The superior length of PacBio HiFi reads supports unambiguous anchoring of the telomeric repeats to each chromosome end. In previous studies, total repeat abundance was reported without linking repeat location to telomeres in general, let alone to individual telomeres [24], or the focus was on only one chromosome end [23]. The superior read length mitigates the challenges arising from having multiple canonical repeats embedded within the variant repeat array (Fig. 2a), which can otherwise be taken as an erroneous indication of the chromosome end [17]. The number of variant patterns detected in our study reached saturation with the 69^th^ accession of the 74 accessions (Fig. S4). Therefore, we were able to detect not only mutation types of high frequency such as TTCAGGG [8], but also a much broader range of variant patterns that is likely to provide a near-complete inventory of variant types. Of note, our results overturn the previous conclusion that there are no variants at the two chromosome ends that cap the large 45S rDNA repeat arrays [38]. In addition, we newly discovered higher-order repeats (HORs), which have before only been described in other satellite regions of A. thaliana such as the centromere [31]. Regarding inter-chromosome similarity of the unique sequence and its subsequent telomeric tract (Fig. S6c), the only other similar example that we are aware of comes from Caenorhabditis elegans [45]. Thus, our work greatly extends our knowledge of telomere-adjacent sequence variation up to the canonical array in this species.*

2. What is the sequencing depth of PacBio datasets utilized? The sequencing depth can influence the repeat number variations reported in different accessions. Is the repeat number variation normalized for the sequence depth?

*Reply: Thank you for your questions!*

*The genome-wide sequencing depth of these 74 accessions ranges from 16 to 212, with a median value of 62. We have now included depth information for each accession in Table S1.*

*From Fig. 3 and Fig. S8, one can see that the length of the variant portion appears to be always normally distributed, regardless of the sequencing depth, and that the degree of variation is not affected by sequencing depth.*

*Our original strategy was to use two random candidate reads from each chromosome end to generate a consensus sequence. The feedback from this reviewer made us realize that we must not ignore the possibility of inadvertently choosing a read with anomalous length. Therefore, we modified our approach to use two reads with median length to generate the consensus sequence. We have revised the Methods section as follows*

*To visualize the degenerate and variant repeat arrays, consensus sequences were generated from two random reads with the median length of the repeat array for each accession and each chromosome end.*

3. It is still possible that a large fraction of telomeric repeats is homogeneous, and only a small fraction present near the transition zones contains high variations. What is the total length of telomeric arrays in Arabidopsis? Is it possible that the authors are capturing only telomeric repeat arrays adjacent to the anchoring reads toward chromosomal arms? A bottom-up approach is needed to determine the total number of telomeric repeats. Next, the fraction of telomeric repeats mapping to the anchoring regions should be determined based on this total number of repeats. This will provide a comprehensive view of canonical and variant telomeric repeat fractions.

*Reply: Thank you for your questions!*

*According to previous studies, the telomere length in A. thaliana accessions ranges from 1 to 12 kb (Choi et al. 2021; Fulcher et al. 2015; Shakirov and Shippen, 2004). In our study, the median value of the total length of the telomeric region (i.e. the entire array of degenerate, variant to canonical repeats) in the candidate reads varies from 745 bp (chr3q of T850) to 10,650 bp (chr5p of IP-Fel-2).*

*As we explained in the manuscript, our focus is on the degenerate and variant telomeric repeat region. For these sequences, we are confident that we have described all variant repeats until the very end, because our strategy is to first split the sequence by repeat unit, examine all repeats until the end, and then visualize from the first degenerate repeat to the last variant repeat. We stop only when we are certain that all subsequent repeats are canonical. This method ensures that we detect all variant repeats in reads and that we do not miss cases like chr3q of Ler-0 (Fig. 2a), where variant repeats occur again after a long canonical repeat array.*

*We did not analyze telomere repeat number or telomere length because standard sequencing approaches (without anchoring one end by adapter ligation (Karimian et al. 2024; Tham et al. 2023; Sholes et al. 2021)) cannot accurately measure the length of telomeres, but only provide a lower limit of telomeric array length. In other words, we cannot know whether the end of a read is the result of random shearing in the telomeric arrays or representing the real telomere end.*

*The region used for anchoring does not include telomeric repeats at all, but rather the repeat-adjacent region (Fig. S2a). In response to the reviewer’s concern, we calculated the fraction of the canonical repeat array and the fraction of non-canonical (degenerate and variant) repeat array for each read. Their lengths show no significant relationship across accessions (Reviewer Figure 7). We visualized four random accessions to provide a more direct representation. The lengths of degenerate and variant repeat region are stable within chromosome ends, regardless of the length of the canonical repeat array measured from reads (Reviewer Figure 8).*

***Reviewer Figure 7.*** *Scatter plot comparing* *the length of the canonical repeat array and the length of degenerate and variant repeat array in individual reads.*

***Reviewer Figure 8.*** *Bar plot showing the length of canonical and non-canonical (degenerate and variant) repeat arrays for each non-rDNA chromosome end in four accessions. Each vertical bar represents a single HiFi read.*

4. The work does not explore the functional consequences of the telomeric variation at the chromatin level or telomeric maintenance perspective in any capacity.

*Reply: Thank you for the comment!*

*Regarding chromatin aspects, we have attempted to detect the methylation status of specific variant types, particularly TTTCGGG, where an A to C substitution at the fourth position creates a CG environment. We attempted to use primrose (*[*https://github.com/mattoslmp/primrose*](https://github.com/mattoslmp/primrose)*) and ccsmeth (*[*https://github.com/PengNi/ccsmeth*](https://github.com/PengNi/ccsmeth)*) for this purpose. However, we found that relying solely on HiFi reads is not precise enough for describing methylation at specific sites yet; it can only illustrate the methylation status in larger regions and will therefore not be an advance over the observations from Farrell et al. (2022).*

*Regarding telomeric maintenance, Reviewer #1 (major concern 1) voiced the same concern. We agree that the proposal of telomerase-independent mechanism is not well supported, and we have therefore removed the related sentences throughout the manuscript.*

4. The authors describe non-repeat sequences within telomeric repeat arrays. However, all three types of these so-called non-repeat arrays are actually repeats—rDNA, higher-order repeats, and repeat variants. Therefore, referring to these sequences as non-telomeric repeats or repeat variants would be more appropriate.

*Reply: Thank you for pointing this out. We agree with the reviewer’s comment. We have replaced the term with “non-telomeric sequences” throughout the manuscript.*

Minor comment: What is the significance of the transition zone with degenerate repeats?

*Reply: Thank you for your question. According to previous studies, the transition zone acts as a bridge between telomeres and chromosome arms (Churikov and Price, 2007). Methylation gradually shifts from an unmethylated status at telomeres to a methylated status towards the centromere side through the transition zone (Farrell et al. 2022; Vrbsky ey al. 2010).*

***Reference***

*Cheng, Haoyu, Gregory T. Concepcion, Xiaowen Feng, Haowen Zhang, and Heng Li. 2021. “Haplotype-Resolved de Novo Assembly Using Phased Assembly Graphs with Hifiasm.” Nature Methods 18 (2): 170–75.*

*Dubocanin, Danilo, Adriana E. Sedeno Cortes, Jane Ranchalis, Taylor Real, Ben Mallory, and Andrew B. Stergachis. 2022. “Single-Molecule Architecture and Heterogeneity of Human Telomeric DNA and Chromatin.” bioRxiv.* [*https://doi.org/10.1101/2022.05.09.491186*](https://doi.org/10.1101/2022.05.09.491186)*.*

*Farrell, Colin, María I. Vaquero-Sedas, María D. Cubiles, Michael Thompson, Alejandro Vega-Vaquero, Matteo Pellegrini, and Miguel A. Vega-Palas. 2022. “A Complex Network of Interactions Governs DNA Methylation at Telomeric Regions.” Nucleic Acids Research 50 (3): 1449–64.*

*Fulcher, Nick, Astrid Teubenbacher, Envel Kerdaffrec, Ashley Farlow, Magnus Nordborg, and Karel Riha. 2015. “Genetic Architecture of Natural Variation of Telomere Length in Arabidopsis Thaliana.” Genetics 199 (2): 625–35.*

*Karimian, Kayarash, Aljona Groot, Vienna Huso, Ramin Kahidi, Kar-Tong Tan, Samantha Sholes, Rebecca Keener, et al. 2024. “Human Telomere Length Is Chromosome End-Specific and Conserved across Individuals.” Science 384 (6695): 533–39.*

*Levenshtein VI. Binary codes capable of correcting deletions, insertions, and reversals. Sov Phys Dokl. 1966;10:707-710.*

*Shakirov, Eugene V., and Dorothy E. Shippen. 2004. “Length Regulation and Dynamics of Individual Telomere Tracts in Wild-Type Arabidopsis.” The Plant Cell 16 (8): 1959–67.*

*Sholes, Samantha L., Kayarash Karimian, Ariel Gershman, Thomas J. Kelly, Winston Timp, and Carol W. Greider. 2022. “Chromosome-Specific Telomere Lengths and the Minimal Functional Telomere Revealed by Nanopore Sequencing.” Genome Research 32 (4): 616–28.*

*Tham, Cheng-Yong, Laifong Poon, Tingdong Yan, Javier Yu Peng Koh, Muhammad Khairul Ramlee, Vania Swee Imm Teoh, Suihan Zhang, et al. 2023. “High-Throughput Telomere Length Measurement at Nucleotide Resolution Using the PacBio High Fidelity Sequencing Platform.” Nature Communications 14 (1): 281.*

*Vrbsky, Jan, Svetlana Akimcheva, J. Matthew Watson, Thomas L. Turner, Lucia Daxinger, Boris Vyskot, Werner Aufsatz, and Karel Riha. 2010. “siRNA-Mediated Methylation of Arabidopsis Telomeres.” PLoS Genetics 6 (6): e1000986.*

**Second round of review**

**Reviewer 1**

I would like to begin by congratulating the authors on a revised manuscript that has addressed the majority of reviewers' comments in a constructive manner. From my perspective, the manuscript has been significantly enhanced and is now much more accessible. The authors expanded the sample size and performed extra analysis to illustrate the genetic groups, the authors also demonstrated that saturation of unique repeats was reached by the 69th accession, which helps to enhance the representativeness and reliability of the conclusions. The figures of main text are also improved.

Some comments that I think are still worthwhile to address:

1. The figures in response lack the necessary annotation (Reviewer Figure 1; Reviewer Figure 2; Reviewer Figure 3; Reviewer Figure 4). The legend indicates the chromosome ends corresponding to the colour of each dot. However, it is also crucial to consider the lines between the dots, which convey additional information. The presence or absence of connections between the dots is also significant. Also, there's something interesting in these figures, as I understand it now the sparseness and denseness of these dots can represent the level of similarity of different telomeric reads from each non-rDNA chromosome end in each accession. Some accessions have a high concentration of reads of each chromosome end (11C1, IP-Bus-0, IP-Fel-2, and IP-Tri-0), while some accessions with reads from some chromosome ends that are very scattered (1q of Ey15, 5q of Had-6b, 5p of Cas-0). What factors are responsible for this variation between different chromosome ends in different accessions?

2. In Figure 2b, the relationships between Europe, Asia, and North America might be made clearer if the corresponding intervals were zooming-in, the scale range of PC2 were adjusted, or PCA were replaced with t-SNE analysis.

**Reviewer 2**

The authors have addressed my concerns in the revised version.

**Authors Response**

**Point-by-point responses to the reviewers’ comments:**

**Reviewer #1:**

I would like to begin by congratulating the authors on a revised manuscript that has addressed the majority of reviewers' comments in a constructive manner. From my perspective, the manuscript has been significantly enhanced and is now much more accessible. The authors expanded the sample size and performed extra analysis to illustrate the genetic groups, the authors also demonstrated that saturation of unique repeats was reached by the 69th accession, which helps to enhance the representativeness and reliability of the conclusions. The figures of main text are also improved. Some comments that I think are still worthwhile to address:

*Reply: We thank the reviewer for the positive feedback!*

The figures in response lack the necessary annotation (Reviewer Figure 1; Reviewer Figure 2; Reviewer Figure 3; Reviewer Figure 4). The legend indicates the chromosome ends corresponding to the color of each dot. However, it is also crucial to consider the lines between the dots, which convey additional information. The presence or absence of connections between the dots is also significant. Also, there's something interesting in these figures, as I understand it now the sparseness and denseness of these dots can represent the level of similarity of different telomeric reads from each non-rDNA chromosome end in each accession. Some accessions have a high concentration of reads of each chromosome end (11C1, IP-Bus-0, IP-Fel-2, and IP-Tri-0), while some accessions with reads from some chromosome ends that are very scattered (1q of Ey15, 5q of Had-6b, 5p of Cas-0). What factors are responsible for this variation between different chromosome ends in different accessions?

*Reply: Thank you for asking this question!*

*The lines between the dots represent read pairs that meet our filtering criteria after performing an all-versus-all alignment. These pairs are recognized as having high similarity.*

*To generate the figures, we first performed an all-versus-all alignment on the reads using minimap2, which produced a PAF file. We then applied a tool from our lab (available on github:* [*https://github.com/Wenfei-Xian/TIPP/blob/main/src/TIPP_telomere.pl*](https://github.com/Wenfei-Xian/TIPP/blob/main/src/TIPP_telomere.pl)*) to filter the PAF file. The output, containing read IDs of the connected pairs, was finally used in igraph (*[*https://igraph.org/*](https://igraph.org/)*) for visualization with the default algorithm - the Fruchterman-Reingold algorithm.*

*The key parameters for determining which read pairs to retain are in lines 133-177 of the script on the github page. Specifically:*

*In line*

*if( $array[9]/$array[10] > 0.98  && $array[1] >= $minimum_uniq && $array[6] >= $minimum_uniq && $array[0] ne $array[5] && $num_X < ($num_I+$num_D) && $max_I < 15 && $max_D < 15 && $num_X < 2*($array[10]/1000) ){*

*0.98 is the substitution percentage threshold, 15 is the maximum indel length, and 2 is the number of substitution per kilobase.*

*In addition, in line*

*if( ($array[2] == 0 && $array[7] == 0 && $array[8]+200 < $array[6]) || ( $array[1] == $array[3] && $array[6] == $array[8] && $array[7] > 200 ) || ( ($array[7] > 200) && ($array[8]+200 > $array[6] )) ){*

*and line*

*if( ($array[2] == 0 && $array[7] == 0 && $array[3]+200 < $array[1]) || ( $array[1] == $array[3] && $array[6] == $array[8] && $array[2] > 200 ) || ( ($array[2]>200) && ($array[3]+200 > $array[1] )) ){*

*200 is the maximum allowable unmapped length, starting from the blunt end after the canonical array has been removed.*

*One limitation of this tool is that it only removes the canonical repeat arrays from reads when performing the all-versus-all alignment, keeping the rest of each read, including the degenerate and variant repeat arrays. As a result, even if the first 3.5 kb of the repeat-adjacent sequences we used to identify candidate reads are identical across all reads, some reads will have less* *similarity with others because they are chimeric reads, have long homopolymer error, or have extended repeat arrays, which easily introduce sequencing errors and obscure the mapping start points. Consequently, pairs with such reads do not meet the default parameters and, therefore, do not appear as connected. We use 5p of Cvi-0 as an example (Reviewer Figure 1; Reviewer Figure 9). The outlier dot represents a chimeric read, where the additional sequence causes a high mismatch rate, failing to pass the filter.*

***Reviewer Figure 9.*** *IGV snapshot of telomeric reads in chr5p of Cvi-0. The red read represents the outlier shown in Reviewer Figure 1, identified as a chimeric read.*

*Regarding the strong concentration of reads in plots for some accessions, such as 11C1, IP-Bus-0, IP-Fel-2, and IP-Tri-0: This results from the exceptionally high genome-wide sequencing depth of these accessions (Table S1). An unusually high number of telomeric reads results in many more pairwise connections at each chromosome end. As a force-directed algorithm to plot the network, the Fruchterman-Reingold algorithm gathers nodes (here, reads) the more connected they are, making them appear more concentrated. When we downsample the coverage of telomeric reads, shown here for 11C1 (from 1,255 to 450) as an example (Reviewer Figure 10a), this effect disappears (Reviewer Figure 10b).*

*Reviewer Figure 10. Clusters of telomeric reads of accession 11C1, as plotted with the Fruchterman-Reingold algorithm. a Result with all 1,255 telomeric reads. b Result with randomly downsampled 450 telomeric reads.*

*The opposite, that the reads from some chromosome ends appear very scattered for some accessions, such as 1q of Ey15 (this should actually be 1p of Ey15-2; we apologize for the mistake in distinguishing the colors of 1p and 1q), 5q of Had-6b, or 5p of Cas-0, is due to the presence of extremely long TTCAGGG variant arrays at these ends (Figure 2a). As discussed above, the default parameter settings for finding connections between reads might be too strict in these cases. Because only connections between very similar sequences are plotted, with longer variant repeat arrays, the likelihood of sequencing errors or true mutations breaking the connections between reads increases, as they are no longer identified as similar. We use 1p of Ey15-2 as an example (Reviewer Figure 11a), loosening the filter parameters from 0.98 to 0.9,* *from 15 to 300, and from 200 to 300 (Reviewer Figure 11b), or removing the variant repeat arrays from reads (Reviewer Figure 11c) before performing an all-versus-all alignment: this results in an increasing number of connections between nodes.*

 ***Reviewer Figure 11.*** *Cluster of telomeric reads from chr1p of Ey15-2.* ***a*** *Result with default filter parameters.* ***b*** *Result with loosened filter parameters.* ***c*** *Result with variant telomeric repeats removed from the reads.*

2.    In Figure 2b, the relationships between Europe, Asia, and North America might be made clearer if the corresponding intervals were zooming-in, the scale range of PC2 were adjusted, or PCA were replaced with t-SNE analysis.

*Reply: Thank you for the comment! We have added a zoomed-in view of the PCA results of the three genetic groups (Europe, Asia, North America) as new figure S8.*

***Figure S8.*** *Zoomed-in view of principal component analysis of European, North American and Asian genetic groups.*

**Reviewer #2:**

The authors have addressed my concerns in the revised version.
*Reply: Thank you!*
